# Elucidating a role for the cytoplasmic domain in the Mycobacterium tuberculosis mechanosensitive channel of large conductance

**DOI:** 10.1038/s41598-018-32536-6

**Published:** 2018-10-01

**Authors:** Nadia Herrera, Grigory Maksaev, Elizabeth S. Haswell, Douglas C. Rees

**Affiliations:** 1 Division of Chemistry and Chemical Engineering 114-96, Howard Hughes Medical Institute, California Institute of Technology, Pasadena, CA 91125 USA; 2 Department of Biology, NSF Center for Engineering Mechanobiology, Washington University in St. Louis, St. Louis, MO 63130 USA; 3 Present Address: Division of Infectious Diseases, Department of Medicine University of California, San Francisco, San Francisco, CA 94143-0654, USA; 4 Present Address: Department of Cell Biology and Physiology, Center for the Investigation of Membrane Excitability Diseases, Washington University School of Medicine, Saint Louis, MO 63110 USA

## Abstract

Microbial survival in dynamic environments requires the ability to successfully respond to abrupt changes in osmolarity. The mechanosensitive channel of large conductance (MscL) is a ubiquitous channel that facilitates the survival of bacteria and archaea under severe osmotic downshock conditions by relieving excess turgor pressure in response to increased membrane tension. A prominent structural feature of MscL, the cytoplasmic C-terminal domain, has been suggested to influence channel assembly and function. In this report, we describe the X-ray crystal structure and electrophysiological properties of a C-terminal domain truncation of the *Mycobacterium tuberculosis* MscL (*Mt*MscLΔC). A crystal structure of *Mt*MscLΔC solubilized in the detergent n-dodecyl-β-D-maltopyranoside reveals the pentameric, closed state-like architecture for the membrane spanning region observed in the previously solved full-length *Mt*MscL. Electrophysiological characterization demonstrates that *Mt*MscLΔC retains mechanosensitivity, but with conductance and tension sensitivity more closely resembling full length *Ec*MscL than *Mt*MscL. This study establishes that the C-terminal domain of *Mt*MscL is not required for oligomerization of the full-length channel, but rather influences the tension sensitivity and conductance properties of the channel. The collective picture that emerges from these data is that each MscL channel structure has characteristic features, highlighting the importance of studying multiple homologs.

## Introduction

**Figure 1:**
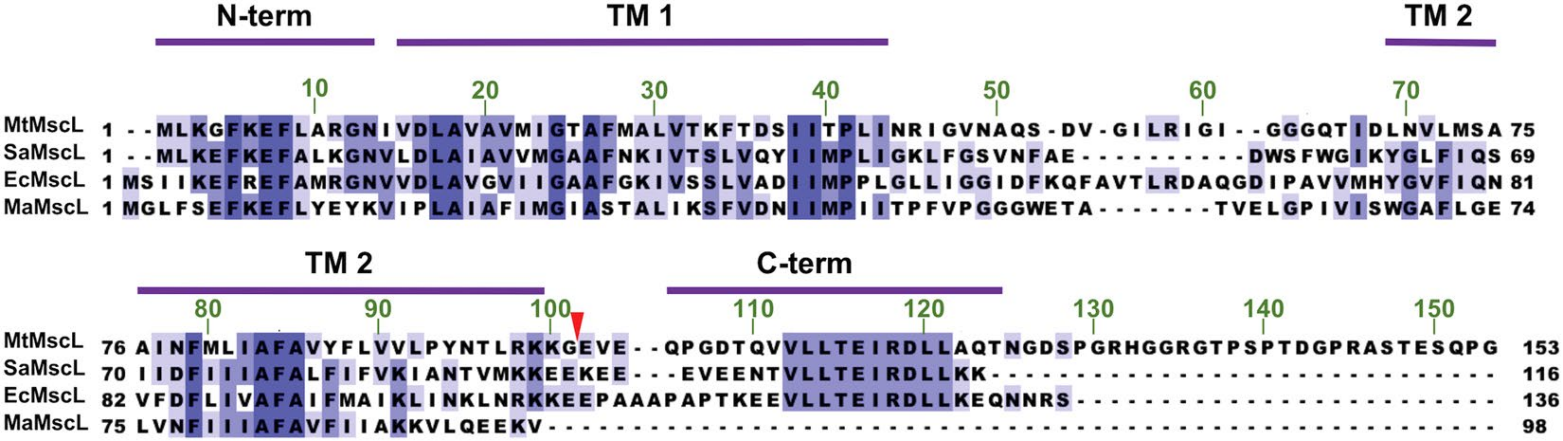
Alignment of MscL sequences. Full-length sequences of *Mycobacterium tuberculosis* MscL, *Staphylococcus aureus* MscL, *Escherichia coli* MscL and *Methanosarcina acetivorans* MscL were aligned using Clustal Omega. The sequence identities between pairs of sequences are *Mt*MscL to *Sa*MscL 40.5%, *Mt*MscL to *Ec*MscL 35.2%, and *Mt*MscL to *Ma*MscL 29.2%. The red triangle over the sequence alignment depicts the location of the (C) terminal truncation after residue 101 of *Mt*MscLΔC. The blue shadings represent sequence identity gradients. The locations of the secondary structure regions are labeled based on the structure for *Mt*MscL, PDBID 2OAR. The residue number legend in green along the top of the alignment corresponds to *Mt*MscL, while the residue numbers on either side of the sequences correspond to that particular homolog. High variability is evident in the periplasmic loop among the homologs.

Mechanosensitive (MS) channels transduce mechanical stimuli into a variety of cellular responses. MS channels are widely distributed through all kingdoms of life, including bacteria and archaea, where they play a prominent role in maintaining proper osmoregulation^1–5^. Sudden changes in osmolarity, such as osmotic downshock during exposure to hypotonic conditions, increases turgor pressure across the cell membrane. This in turn increases the tension within the lipid bilayer and activates a network of MS channels which respond by opening typically nonselective pores in the membrane to release cell contents and alleviate the pressure^1,5–7^. Bacterial MS channels are characterized by their relatively high and non-selective conductances, ranging from ~0.1 nS to ~3 nS under defined experimental conditions^6–8^, which is up to several orders of magnitude greater than typical for ion-selective channels^3,6,9^. Of these channels, the mechanosensitive channel of large conductance (MscL) exhibits the largest conductance, with an estimated open state pore diameter of ~25–35 Å - large enough for the passage of molecules up to 9 kDa^4,7,10,11^. In view of the high conductance yet small subunit size of MscL (*Escherichia coli* MscL (*Ec*MscL) has 136 residues; Fig. 1), the oligomeric nature of this channel was appreciated from its initial discovery^11^. Considerable efforts have been made to establish the molecular mechanism of MscL gating^3,12^, employing a variety of techniques including electrophysiology, biochemical and genetic studies, molecular dynamics simulations, and X-ray crystallography. Interest in understanding the factors controlling the high tension sensitivity and conductance of MscL has been heightened by reports utilizing this channel as a tension sensitive element in nanodevices and drug delivery systems, as well as a role for MscL in the uptake of antibiotics by bacteria^13–16^.

**Figure 2:**
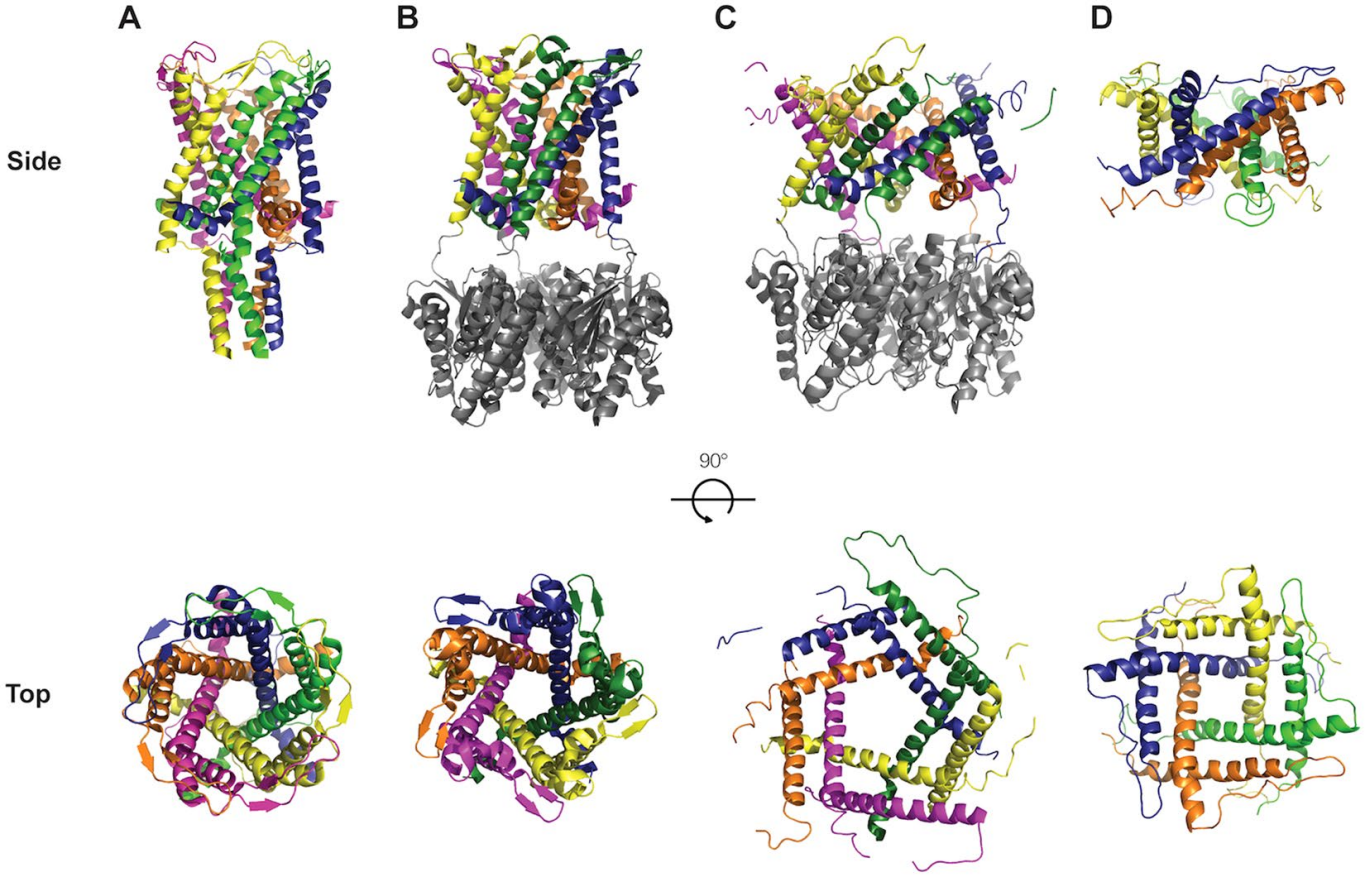
Crystal structures of MscL. Side and top views of the available crystal structures of MscL in a ribbon diagram, depicting (**A**) native (full length) *Mt*MscL PDBID 2OAR, (**B**) *Ma*MscL closed PDBID 4Y7K, (**C**) *Ma*MscL expanded PDBID 4Y7J, and (**D**) tetrameric *Sa*MscLΔC PDBID 3HZQ. The individual subunits in each structure are colored separately. Side views in panels (**B**) and (**C**) include the MjRS protein (colored in grey) that was fused to *Ma*MscL for crystallization. The top row corresponds to a side view with the periplasmic side of the proteins facing up and the cytoplasmic side of the proteins facing down. The bottom row corresponds to a top periplasmic view of the channels.

The first crystal structure determination of MscL was obtained for the *Mycobacterium tuberculosis* homolog (*Mt*MscL), solved to 3.5 Å resolution^17,18^. This structure revealed a pentameric channel with a relatively simple subunit architecture consisting of a cytoplasmic N-terminal amphipathic helix, two transmembrane helices (TM1 and TM2) connected by a periplasmic loop, and a cytoplasmic C-terminal helix (Fig. 2). Several key structural features, including TM1 helices that form the permeation pathway and TM2 helices that form the channel–membrane interface, have been observed in the subsequently determined MscL structures from *Staphylococcus aureus* (*Sa*MscL^19^) and *Methanosarcina acetivorans* (*Ma*MscL^20^) (Fig. 2). The assembled channel can be considered to be composed of two major elements: the membrane spanning region that forms the high conductance permeation pathway in the open state, and the C-terminal cytoplasmic region. The narrowest opening of the permeation pathway in the *Mt*MscL crystal structure was found to be ~3 Å, and formed through a constriction provided by the sidechains of Ile 17 and Val 21 from TM1. Since the minimum diameter required for the passage of water or ions through hydrophobic pores is calculated to be 9 and 13 Å^21^, respectively, the *Mt*MscL structure was classified as a non-conducting (closed) conformation. Estimates of the open state diameter range from ~25–35 Å based on the large conductance and the sizes of molecules that can pass through the open channel^10,22^. These conclusions are supported by FRET observations^23^, and models for the open state have been developed through modeling and molecular dynamics simulations^24,25^. To date, however, the structure of the open state of MscL remains elusive.

While the membrane spanning region of MscL serves as the tension sensor and provides the gated permeation pathway, the role of the C-terminal domain is not as well understood. One challenge in defining a role for the C-terminal domain is that, while the sequence corresponding to the cytoplasmic helix is generally conserved, MscL function is largely insensitive to mutagenesis^26,27^ or truncation^19,28,29^ of the C-terminal region. Indeed, MscL homologs exist that lack this entire domain such as *Ma*MscL (Fig. 1). Potential roles for the C-terminal domain include regulating the oligomeric state of the channel and/or tuning the mechanosensitivity of MscL^19,30^. Evidence for these functions may be summarized as follows:

(i) A proposed role for the C-terminal domain in defining the MscL oligomeric state was motivated by the observation that a C-terminal truncation of *Sa*MscL (*Sa*MscLΔC) formed a tetramer (Fig. 2D)^19^. The subsequent report that the X-ray structure of the isolated C-terminal domain of *Ec*MscL was pentameric^31^ suggested that the C-terminal domain might itself direct the oligomeric state of the entire MscL channel^17,31,32^. Biochemical and biophysical studies testing this proposal, however, have yielded conflicting results. While *in vitro* studies have demonstrated that removal of the C-terminus can perturb the oligomeric state^33–36^, the strong effect of detergent on the stability of oligomers confounds these analyses. The available data on the *in vivo* oligomeric state are consistent with a pentamer for both full length and truncated versions of *Ec*MscL in the membrane^37^.
(ii) A role for the C-terminal domain in regulating mechanosensitivity is supported by the observation that the *Ec*MscL channel with a C-terminal domain truncation requires slightly higher tensions to gate than the wild type, and fails to exhibit subconducting states during both opening and closing gating events^1,28,29^. This is also observed with *Sa*MscLΔC, which shows more stable gating events than the flickering events observed for full length *Sa*MscL^19,37^. Some molecular dynamic simulations have suggested that the closed-to-open transition is accompanied by dissociation of the C-terminal helix bundle, but this is not universally observed^23,24^. Experimentally, stabilization of the C-terminal helix bundle with disulfide bonds does not impair function^29,30^, indicating that the bundle remains intact during gating, consistent with a thermodynamic analysis of the helix stability of the isolated C-terminal peptide^31^. Truncation of the C-terminal sequence does not significantly perturb the open state conduction, although shortening of the TM2 - helix linker does reduce the conductance^30^.

To generate a more complete picture of the role of the C-terminal domain, we endeavored to structurally and functionally characterize the C-terminal domain truncation of *Mt*MscL. To date, *Mt*MscL is the only full-length MscL with a crystallographically determined structure^17^. The crystal structure of *Mt*MscLΔC presented in this work demonstrates that the C-terminal domain is not essential for assembly of a pentameric membrane pore domain, although indirect crystallographic evidence suggests that truncation may perturb the equilibrium between oligomeric states. Electrophysiological characterization in *E. coli* spheroplasts demonstrates that while *Mt*MscLΔC remains mechanosensitive, it exhibits both a higher conductance and a higher tension sensitivity when compared to full-length *Mt*MscL. Our results thus establish that while the C-terminal domain is not necessary for forming a functional state of *Mt*MscL or mechanosensitivity, it does influence channel properties.

## Results

### The effects of C-terminal truncation on the structure of *Mt*MscL

**Figure 3:**
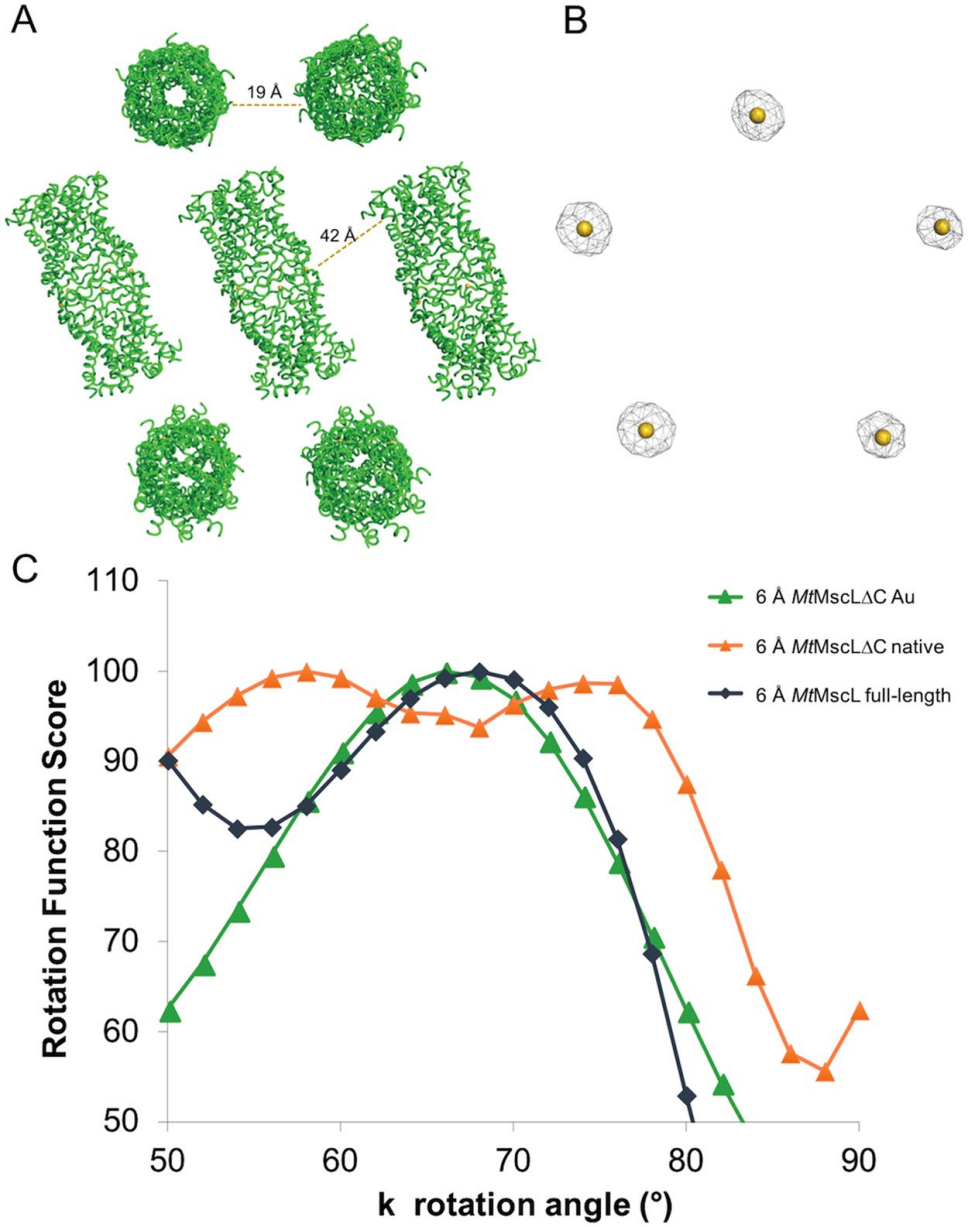
Crystallographic analysis of *Mt*MscLΔC. (**A**) Crystal packing of *Mt*MscLΔC, depicting the head to head packing arrangement between adjacent pentamers. The distances of closest approach between channels along the different lattice directions are indicated; as they are too long for protein-protein contacts they presumably must be mediated at least in part by detergent micelles. (**B**) Anomalous difference Fourier map calculated at 6 Å resolution of the Au derivative using SAD phases reveal the pentameric arrangement (**C**) An analysis of self rotation functions as a function of the κ rotation angle about the direction of the 5-fold axis for *Mt*MscLΔC Au data (green), *Mt*MscLΔC native crystals (orange) and full length, wildtype MtMscL 2OAR (dark blue). The data were analyzed with a 6 Å resolution cutoff.

After screening several constructs, a variant of *Mt*MscL was identified that expressed and purified well. This construct, truncated after residue 101 (Fig. 1, red triangle), was therefore selected for further analysis and is hereafter referred to as *Mt*MscLΔC. Although this construct crystallized readily, the crystals typically diffracted poorly, to ~9 Å resolution. After screening ~2000 crystals, a crystal was found that diffracted to ~5 Å resolution. The diffraction data collected from this crystal was used to initially solve the structure by molecular replacement, using as the search model the pentameric *Mt*MscL full-length structure with the C-terminal domain computationally truncated. Using this search model in Phaser MR, a TFZ score of 17.7 and an LLG score of 304.1 were obtained, supporting the validity of the molecular replacement solution. The asymmetric unit for this solution consisted of two pentameric channels, corresponding to a Matthews coefficient of 5.0 Å^3^/Dalton and a ~75% solvent content. The major crystal contacts were mediated by the head-to-head association of two pentamers through the periplasmic loops such that five-fold axes were coincident; similar contacts were observed in the full-length *Mt*MscL structure (Fig. 3A). The remaining contacts required to form a three-dimensional lattice were not readily identified, as the minimal separations between adjacent pairs of pentamers are ~19 Å, precluding obvious contacts involving protein-protein interactions (Fig. 3A). This suggests that contacts between detergent micelles, rather than protein, mediate lattice formation along these directions, plausibly contributing to the relatively poor overall diffraction quality.

To verify the molecular replacement data described above, a heavy atom derivative was prepared with sodium aurothiosulfate. This gold compound was used in the original *Mt*MscL structure determination and was found to bind to the periplasmic loops at the crystal contact between adjacent pentamers. A dataset was collected just above the Au L-II edge at 13815 eV and processed to 5.8 Å resolution. The single wavelength anomalous diffraction (SAD) data were used to search for the heavy atom positions using molecular replacement - SAD phasing in the Phaser program from the Phenix crystallographic programming suite^38,39^. These results identified 5 gold sites sandwiched between the periplasmic loops of two adjacent pentamers, at the same locations observed in the full-length protein (shown in Fig. 3B). To minimize model bias, SAD phases were calculated from the Au data alone with Phaser. These phases were improved using the CCP4 program DM to iteratively 10-fold average the electron density over the two pentamers related by non-crystallographic symmetry. The resulting electron density revealed the permeation pathway for *Mt*MscLΔC to be in the closed conformation, with density covering TM1 and the N-terminal part of TM2, although the individual helices were not defined.

Crystallographic structure refinement was challenging, undoubtedly due to the low resolution and associated high temperature factors. Additionally, a rotation function analysis suggested heterogeneity in the crystal packing (Fig. 3C), possibly due to variability in the rotational orientation of pentamers around the 5-fold axis, or to the presence of multiple protein oligomeric states in the crystal. The self-rotation function calculated from the diffraction data obtained from the gold derivatized *Mt*MscLΔC crystal (Supplementary Fig. S1) exhibits characteristic features of 522 point group non-crystallographic symmetry, with a 5-fold axis peak in the κ = 72° section near (ϕ, ω) = (69°, 54°) and a series of prominent peaks in the corresponding plane perpendicular to that axis in the κ = 180° section. The 5-fold peak arises from the pentameric arrangement of *Mt*MscLΔC, while the perpendicular two-fold axes are generated from the non-crystallographic symmetry arising from the two pentamers packed in head-to-head fashion. In the native data set (Supplementary Fig. S2), however, the five-fold peak is broader and the two-fold section, while prominent, no longer has well defined peaks. This behavior suggested variability in either the orientation or the oligomeric state of *Mt*MscLΔC in the native crystal as noted above.

To probe this possibility in more detail, the value of the self-rotation function along the channel rotation axis ((ϕ, ω) = (69°, 54°)) was calculated as a function of the rotation angle κ using the CCP4 program Polarrfn (Fig. 3C). The gold data set exhibited a broad peak near κ = 67° that, while displaced from that expected for a five-fold, matches well that observed using the diffraction data for the full length *Mt*MscL structure (Fig. 3C). Interestingly, the native *Mt*MscLΔC data set produced two peaks at κ = 58° and 74°. Both pentamers and hexamers could be present in the crystal, or perhaps that there is a single pentameric state that can vary in orientation around the 5-fold axis. Given the limited resolution of the data sets, it was deemed unlikely that these possibilities could be convincingly resolved. Consequently, the refinement proceeded with the gold data set, since the self-rotation function is indicative of a pentameric state, validated by the location of five Au sites in an anomalous difference Fourier map (Fig. 3B).

**Figure 4:**
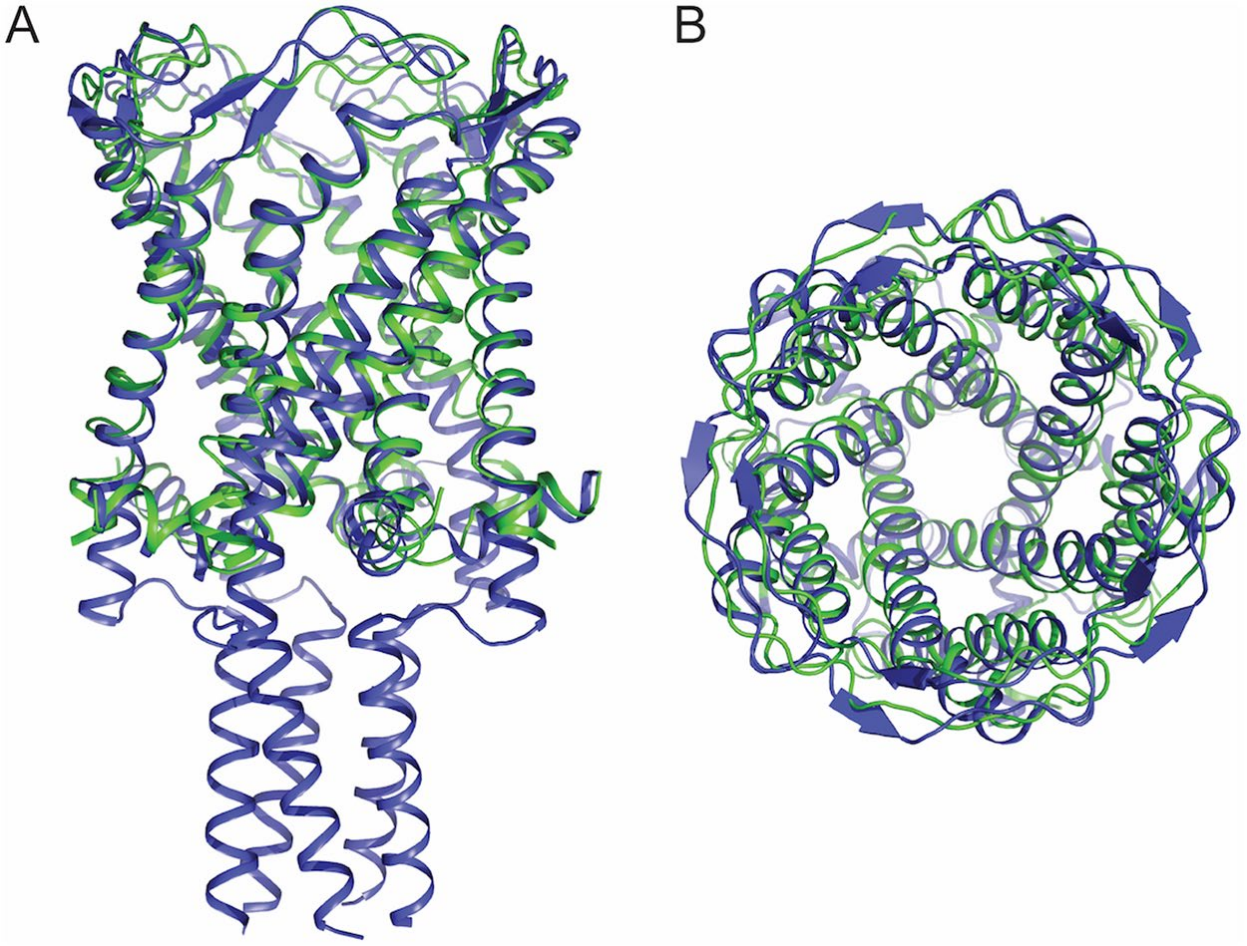
Structural superposition of *Mt*MscLΔC with *Mt*MscL full-length. MtMscLΔC is colored in green and *Mt*MscL full-length is colored in blue. (**A**) Depicts the view parallel, and (**B**) depicts a view perpendicular to the five-fold axis, illustrating the structural similarity of the transmembrane domains in these two structures.

To refine the structure, *Mt*MscL was truncated from residues 102–151 *in silico*; after initial refinement, C-terminal residues 97–101 were removed as they were not resolved in the refined density. The final refinement statistics are R_work_/_free_ = 0.37/0.38 (Table 1). An alignment of *Mt*MscLΔC with full length *Mt*MscL gave a root mean squared deviation (RMSD) in the Cα positions of 1.4 Å, indicating that the structure remained relatively unchanged as a consequence of C-terminal truncation (Fig. 4). Coordinates for *Mt*MscLΔC were deposited to the RSCB Protein Data Bank under PDBID 6CTD.

**Table 1:** Data collection and refinement statistics for *Mt*MscLΔC.

	Sodium Aurothiosulfate	Native
**Data collection**		
X-ray source	SSRL BL 12-2	SSRL BL 12-2
X-ray wavelength (Å)	0.8974	0.9795
Space group	P 2_1_	P 2_1_
Cell dimensions *a, b, c* (Å), β (°)	84.7, 110.3, 136.8, 91.2	86.8, 111.9, 139.4, 92.2
Resolution (Å)^a^	35.0-5.5 (5.5–6.15)	35.0-6.0 (6.0–6.7)
No. of unique reflections	7936	6697
R_meas_^a^ (%)	5.2 (124.7)	3.3 (128.4)
R_merge_^a^ (%)	4.8 (115.8)	3.0 (126.4)
<I/σ I>^a^	12.4 (1.8)	17.7 (1.8)
Completeness (%)^a^	96.0 (98.6)	98.7 (98.5)
<Redundancy>	7.0	6.9
**Refinement**		
Resolution (Å)	23-5.8	—
No. of reflections	6049	—
R_work_/R_free_ (%)	0.37/0.38	—
<B-factor (Å^2^)>	567	—
Bond lengths (Å)	0.007	—
Bond angles (°)	1.12	—
Ramachandran Plot (%)^b^		—
Favored regions	94 (90)	—
Allowed regions	6 (10)	—
PDB ID	6CTD	—

^a^Indicates high resolution parameters presented in parentheses.

^b^Value given by Coot outside of parenthesis; value given by PDB validation report, in parentheses.

### *Mt*MscLΔC has a higher conductance than that of *Mt*MscL, similar to that of *Ec*MscL

**Figure 5:**
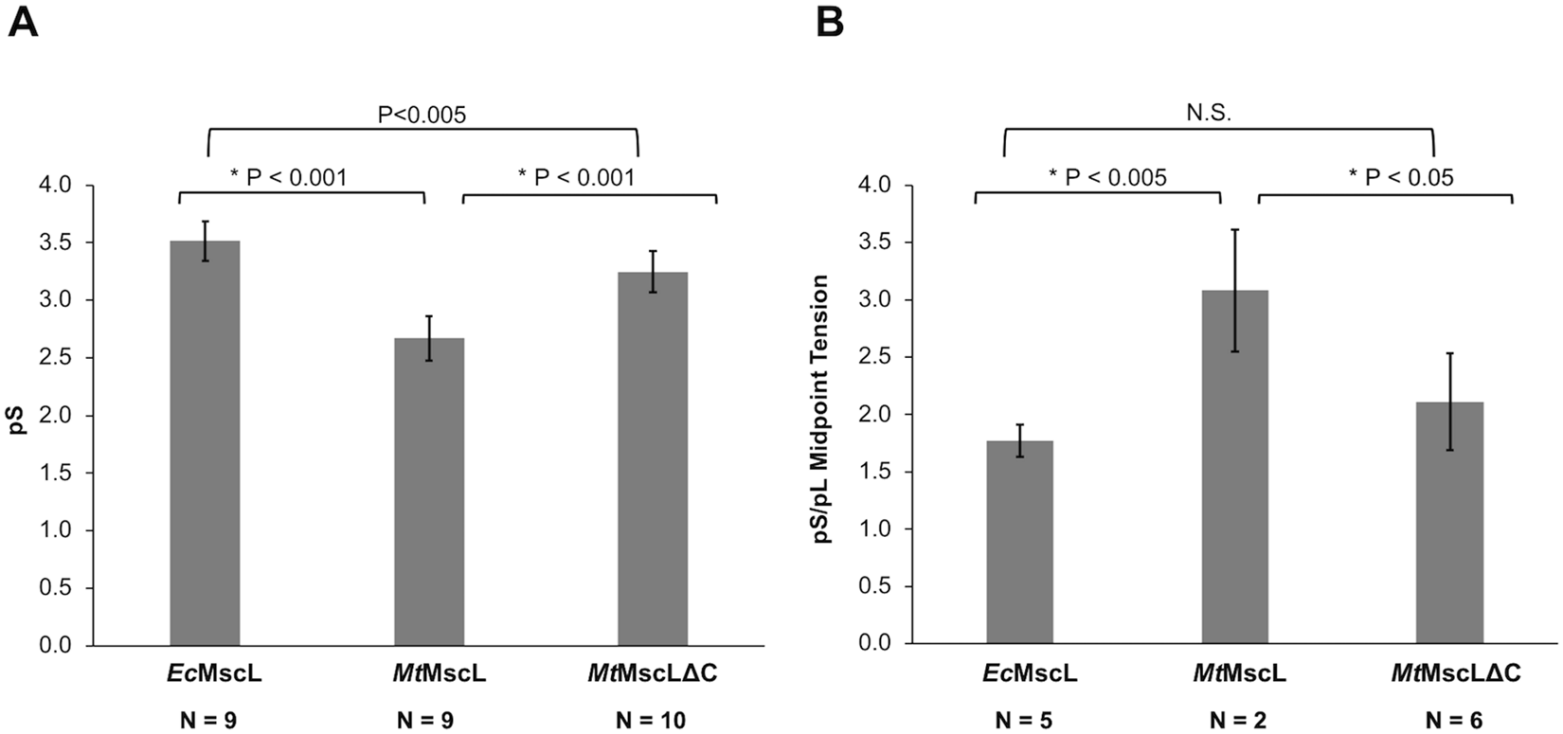
Unitary conductance and MscL/MscS midpoint ratios. (**A**) Average unitary conductance from the indicated number of patches from BL21 DE3 *ΔmscL* cells expressing *Ec*MscL WT, *Mt*MscL WT, or *Mt*MscLΔC. Membrane potential was clamped at −20 mV. (**B**) MscL/MscS midpoint ratios for *Ec*MscL, *Mt*MscL WT, and *Mt*MscLΔC from the indicated number of patches derived from BL21 DE3 *ΔmscL* cells. Significant differences between pairs, based on Student’s t-test are reported above the charts.

We used single channel patch clamp electrophysiology to characterize the channel properties of *Mt*MscL and *Mt*MscLΔC expressed in *E. coli*, and to compare them to *Ec*MscL. Giant *E. coli* spheroplasts were generated from BL21 DE3 *ΔmscL* cells expressing *Mt*MscL, *Mt*MscLΔC or *Ec*MscL. Inside-out patches were subjected to a pressure ramp protocol wherein membrane tension is increased in response to suction in the patch pipette. Representative traces from giant spheroplasts of *E. coli* expressing each of the three constructs are shown in Supplementary Fig. S3. The average unitary conductance of *Ec*MscL at −20 mV was observed to be 3.5 ± 0.1 nanoSiemens (nS) (Fig. 5A); this result is in accordance with previous reports^5^. Full length *Mt*MscL exhibited a conductance of 2.7 ± 0.2 nS, while *Mt*MscLΔC had a significantly larger conductance of 3.3 ± 0.2 nS (p < 0.001, Student’s t-test) (Fig. 5A).

To assess the relative tensions required for gating of these three channels, we calculated the midpoint pressure for channel opening relative to the endogenously expressed *Ec*MscS (typically referred to as pL/pS^40^). Midpoint pressures are defined as the pressure where half of the channels of the population under consideration are open. Measurements of relative midpoint pressures therefore require saturation of both the *Ec*MscS and MscL currents. The pL/pS ratios for *Ec*MscL full-length, *Mt*MscL full-length, and *Mt*MscLΔC were 1.63 ± 0.06, 3.04 ± 0.38, and 1.93 ± 0.22, respectively (Fig. 5B). *Mt*MscLΔC gated at a significantly lower tension than *Mt*MscL (p < 0.05, Student’s t-test) while the pL/pS of *Ec*MscL and *Mt*MscLΔC were not significantly different. We note that we were only able to reach current saturation with WT *Mt*MscL before the patch ruptured in 3 experiments out of 12, which is not surprising as *Mt*MscL is reported to gate at the tensions approaching lytic tensions of the patch^41^. Taken together, the data in Fig. 5 indicate that *Mt*MscLΔC more closely resembles *Ec*MscL than full-length *Mt*MscL, both in terms of conductance and tension sensitivity.

### Inferring the conductance of a channel from the structure

The functional state of a channel cannot be directly assessed from the structure, but rather must be indirectly inferred from what is known about the properties of different states. Qualitatively, the conductance of a channel will reflect the geometry of the permeation pathway, with a wider pathway typically viewed as corresponding to a state of greater conductance. As an example, *Mt*MscL and *Mt*MscLΔC are assigned to representing the closed state on the basis of a pathway through the channel which is too narrow to support the high conductances observed in the fully open state. The conductances corresponding to the structures of the expanded states observed for *Sa*MscLΔC and *Ma*MscL are not known. While the permeation pathways in these two structures are too narrow to correspond to the fully open state, they also do not appear to be completely closed in *Sa*MscLΔC and *Ma*MscL expanded states. To address the conformational states available to MscL and their functional significance, a comparative analysis of the permeation pathway geometries of the available MscL structures, including our newly analyzed *Mt*MscLΔC (summarized in Figs 2 and 4) was conducted.

MscL structures may be classified into two major conformational states through an analysis of the crossing angles of the transmembrane helices^19,20^. There are three primary helix-helix interfaces that can be used to describe the MscL structures, namely the crossing angles between TM1s in adjacent subunits (TM1-TM1′), between TM1 and TM2 in the same subunit (TM1-TM2), and between TM1 and TM2 in adjacent subunits (TM1-TM2′). Helix crossing angles for all available MscL structures are presented in Table 2. As noted previously^19^, the TM1-TM2′ contact is conserved in these structures, with a nearly antiparallel crossing angle of ~166° providing an extensive interface. In contrast, the TM1-TM1′ and TM1-TM2 crossing angles fall into two groups, representative of closed (*Mt*MscL, *Mt*MscLΔC and *Ma*MscL closed) and expanded (*Sa*MscLΔC and *Ma*MscL expanded) conformations, respectively (Table 2). The closed conformation is characterized by TM1-TM1′ and TM1-TM2 angles of ~45° and 130°, respectively, while the corresponding values for the expanded state are ~60° and 112°, respectively (Supplementary Fig. S4).

**Table 2:** Transmembrane helix crossing angles and RMSD between MscL channel structures.

	Closed			Expanded	
MscL Homologue and PDBID	*Mt* ∆C 6CTD	*Mt* 2OAR	*Ma*^closed^ 4Y7K	*Sa* ∆C 3HZQ	*Ma*^expanded^ 4Y7J
Crossing Angle TM1-TM2′	170°	166°	158°	169°	164°
Crossing angle TM1-TM2	130°	130°	130°	112°	112°
Crossing Angle TM1-TM1′	43°	43°	47°	65°	57°
RMSD (Å) Subunit A	—	1.0	1.8	6.2	5.7
RMSD (Å) TM helices in pentamers	—	1.4	2.3	—	3.8

The crossing angles between TM1-TM2′, and between TM1-TM2 (in the same subunit) and TM1-TM1′ in different subunits are reported for the MscL structures solved to date. The root mean square deviation (RMSD) between *Mt*MscLΔC and other MscL structures are reported for a single subunit (subunit A), and for all the TM helices in the pentameric MscL structures.

To assess the functional significance of these conformational states, the permeation pathways in the different MscL structures were characterized with the program HOLE^42^, which evaluates pore geometry and estimates the corresponding conductances. For comparison, a reference set of 4 proteins of high (~nS) conductance were also analyzed, three β-barrel proteins (two porins and VDAC) and *E. coli* MscS (*Ec*MscS) in an open conformation^43–46^. Since channel conductances are sensitive to the solution conductivity, the measured conductances for the open state of MscL were approximately corrected to 1 M KCl, the conditions reported for the electrophysiological characterization of the porins, VDAC and *Ec*MscS. The solutions typically used for MscL electrophysiology experiments have conductivities of ~30 mS cm^-1^ ^47^ or ~0.27 times that of 1 M KCl (~110 mS cm^-1^;^48^. Since the conductance of *Ec*MscL does not exhibit current saturation up to 2 M KCl^1^, the MscL conductances were corrected to the corresponding values in 1 M KCl by dividing by 0.27.

**Figure 6:**
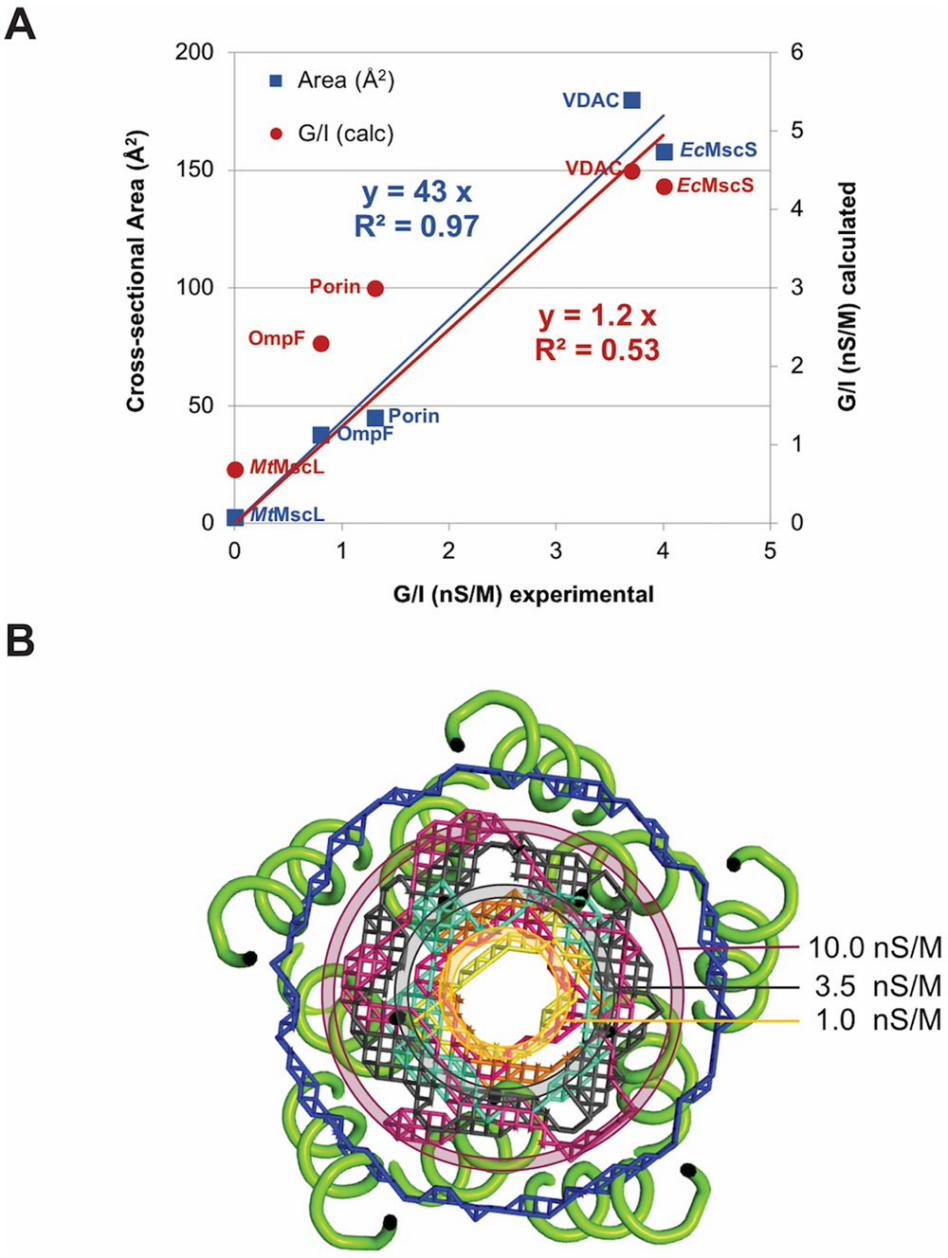
Analysis of pore area in relation to conductance in various MS channels. (**A**) Calibration curve between the experimental conductance, the conductance calculated by HOLE and the minimum cross-sectional area evaluated for four high conductance channels of known structure: OmpF, Porin, MscS, and VDAC. Data for this figure is presented in Table 3, with the point near the origin provided by the values for *Mt*MscL in the closed conformation with a conductance of 0 nS. Linear fits were constrained to pass through the origin. (**B**) Structure of *Mt*MscLΔC with mesh representation of the permeation pathway at the point of greatest restriction for channels of different conductances. Mesh colors: *Ec*MscL model open- blue, *Sa*MscLΔC (3HZQ) magenta (inner), *Ma*MscL expanded (4Y7J)- cyan, MscS open (2VV5)- gray, porin (1PRN)- orange, porin (2OMF)- yellow, VDAC (3EMN) - warm pink (outer). The rings depict pore diameters at the restriction point for channels of indicated conductance, evaluated from equation (3).

For this reference set, the relationship between the conductances calculated with HOLE, G_calc_, and the experimentally observed values, G_obs_ (corrected to 1 M KCl), may be approximated by a linear relationship (Fig. 6A):

1 $${{\rm{G}}}_{{\rm{obs}}}={{\rm{G}}}_{{\rm{calc}}}/1.2$$

While the largest conductance channels (VDAC and EcMscS) are closely replicated by the HOLE calculation, the conductances for the porins are overestimated as previously observed^42^; these effects contribute to a modest R^2^ value for the fit (0.53). Using this calibration curve, the conductances of the conformations of the expanded *Sa*MscLΔC and *Ma*MscL structures determined crystallographically are estimated as ~ 2–3 nS M^−1^.

The permeation pathways of these channels have large openings at one or both membrane surfaces tapering to a relatively narrow region and hence may be approximately described as funnel or hour-glass shaped. As a result, the overall channel conductance will be dominated by this constriction when an Ohm’s law-type model is used (see^47^). Consequently, the HOLE calculation was supplemented with an estimate of the cross-sectional area at the constriction point, as approximated by the number of grid points in a plane approximately perpendicular to the permeation pathway (Table 3). For the reference set, the minimum cross-sectional area, A (in Å^2^), and the experimentally determined specific conductances (G_obs_, the conductance in nS corrected to 1 M KCl) are empirically related by the linear expression (Fig. 6A):

2 $${\rm{A}}=43\,{{\rm{G}}}_{{\rm{obs}}}$$

**Table 3:** Conductance and permeation pathway properties of MscLs and calibration channels.

Protein	PDB	Ref.	G_obs_ nS (Crystal structure)	G/I (obs) nS M^−1^	G/I (calc) nS M^−1^ (HOLE)	Area (Å^2^) (at pore constriction)	Calc G/I nS M^−1^ (Eq. 2)
**MscL Structures**							
*Mt*MscL closed	2OAR	^17^This work	0	0	0.7	3	0.1
*Mt*MscLΔC closed	6CTD	This work	0	0	0.7	1	0.0
*Sa*MscLΔC expanded	3HDZ	^19^	—	—	2.2	29	0.7
*Ma*MscL expanded	4Y7J	^20^	0.25 (open)	0.9	3.5	85	2.0
*Ec*MscL open	model	^49^	3.5	13.0	31	604	14.0
**Calibration Channels**							
VDAC	3EMN	^45^	3.7	3.7	4.5	180	4.2
OmpF	2OMF	^54^	0.8	0.8	2.3	38	0.9
Porin	1PRN	^44^	3.9 (per trimer)	1.3	3.0	45	1.0
*Ec*MscS Open	2VV5	^46, 48^	4.0	4.0	4.3	158	3.7

The observed and calculated conductances are tabulated for MscLs of defined structure, along with nS conductance calibration channels. The observed conductances for the open states of the corresponding channels, G(obs), are corrected for the ionic strength of the conductance measurements (eff. I (M)) to yield the G/I (obs) for the open state structures, reported in the concentration independent units of nS M^−1^ (‘G/I (obs) nS M^−1^’ column). The conductances calculated by the default mode of HOLE^42^ are listed in the ‘G/I (calc) nS M^−1^ (HOLE)’ column, along with an estimate of the conductance derived from the cross section area at the pore constriction, using the empirical parameterization provided in Eq. 2 (‘Calc G/I ns M^−1^ (Eq. 2)’ column). The relationships between the observed, HOLE-derived conductances, and the cross-sectional area at the pore constriction are depicted in Fig. 6A.

This fit is improved relative to equation (1) (R^2^ = 0.97), with an estimated standard deviation for the linear coefficient of 43 ± 2. The effective pore radius at the constriction point of a channel of specified conductance can be extracted from the cross-sectional area assuming a circular geometry:

3 $${\rm{R}}={(43{{\rm{G}}}_{{\rm{obs}}}/\pi )}^{1/2}$$

The equivalent radii, R, of circular pores corresponding to conductances of 1, 3.5 and 10 nS M^−1^, (3, 7.0 and 12 Å, respectively) are illustrated in Fig. 6B and approximate the observed conductances of porins, and the open states of MscS and MscL, respectively. From this empirical calibration curve, the corresponding conductances of the available MscL structures and the computational model for the open state^49^ may be estimated (Table 3). The structures observed for *Mt*MscL and *Mt*MscLΔC are expected to exhibit minimal conductance, consistent with the assignment of these conformations to the closed state. More surprisingly, the expanded conformations observed in the crystal structures of *Sa*MscLΔC and *Ma*MscL are calculated to have conductances of ~1 nS M^−1^ (0.7 and 2.0 nS M^−1^ respectively) or ~0.3 nS under the typical conditions used for MscL electrophysiology. While this is well below the ~10 nS M^−1^ observed for the fully open conformation of *Ec*MscL or *Mt*MscL, it is close to that observed for *Ma*MscL (0.25 nS^20^). Thus, this expanded conformation observed for *Ma*MscL and *Sa*MscLΔC may correspond to a sub-conductance state of other MscL channels.

## Discussion

The crystal structure of *Mt*MscLΔC reported in this paper establishes that removal of the C-terminal sequence after residue 101 has little consequence for the overall structure of the membrane domain relative to *Mt*MscL. While many MscL homologs have been shown to maintain a pentameric state in DDM, the detergent used to purify and crystallize *Mt*MscLΔC^12,28,33^, changes in oligomeric state have been observed when using alternate detergents. In particular, when using C8E4 and LDAO^34,36^, mass spectrometry studies of MscL homologs^34^ and the crystal structure analysis of *Sa*MscLΔC show alternate oligomeric states^19^. Indeed, there is also evidence in our crystallographic analysis that oligomeric states other than a pentamer may be present (Fig. 3C), although this could not be definitively established.

The conformational space of MscL has been crystallographically explored through the use of different homologs, detergents and constructs. The two states of MscL that have been characterized to date correspond to closed (such as *Mt*MscL full length and *Mt*MscLΔC) and expanded conformations (such as *Sa*MscLΔC and *Ma*MscL expanded), where the latter likely corresponds to a sub-conductance state. Yet to be structurally captured is the fully open state of MscL, which in the absence of applied tension should be of sufficiently higher energy than the closed state to prevent the spontaneous opening of the channel. The challenge for the structural characterization of MscL is to identify conditions where the fully open state is stable and amenable to high resolution structural studies, either by crystallography or, as seems increasingly likely, the single particle cryo-electron microscopy methods which are revolutionizing structural biology.

We have shown that the function of *Mt*MscL expressed in *E. coli* BL21 DE3 *∆mscL* cells can be analyzed by patch clamp electrophysiology, in accordance with the methodology used on the recent study of *Ma*MscL^20^. This expression system allowed us to increase the amount of protein expressed in the cell compared to previous methods that utilize a low expression vector, pB10b^41^ and increase our chances of observing a gating event. This was critical for our efforts, particularly when working with *Mt*MscL, which has a shifted activation curve that reaches the lytic limits of *E. coli*. Electrophysiological characterization of *Mt*MscLΔC indicates that its basic properties (unitary conductance and MscL: MscS activation midpoint ratio) are closer to those of *Ec*MscL than *Mt*MscL. Overall, these results support the hypothesis that the C-terminal domain is not essential for the intrinsic mechanosensitivity of *Mt*MscL. The C-terminus, however, clearly plays a role in the detailed electrophysiological characteristics of the channel, in particular modulation of gating tension and conductance. The C-terminal domain of *Mt*MscL could undoubtedly play other functional roles, perhaps interacting with other proteins or serving as a filter to limit the release of cellular contents during gating of MscL^4,50^. If used as filter, it would parallel with the role for the C-terminal domain of *Ec*MscS, where it serves as a pre-filtering domain^51^. In addition, it would shed light on the observed higher conductance and disappearance of sub-conducting states in C-terminal truncations observed in *Ec*MscL and *Sa*MscL, respectively.

Our observation that truncation of the C-terminal domain in *Mt*MscL results in a decreased tension requirement for gating contrasts with previous observations of *Ec*MscL and *Sa*MscL, where C-terminal truncation increases the tension threshold for gating^19,29,52^. The underlying mechanistic basis for these distinctions is not evident. The inescapable conclusion from an analysis of the data available for MscL channels from different species is that despite major structural and sequence similarities across MscL homologs, they do not all function identically, particularly with regards to the role of the C-terminus. Consequently, it is of high importance to study multiple MscL homologs to obtain a balanced view of structure and function relationships, rather than assuming they all look and function similarly.

## Materials and Methods

### Protein cloning, expression, and purification

Using site-directed mutagenesis on a plasmid prepared for previous *Mt*MscL studies^17^, two mutations were introduced to *Mt*MscL in pET-19b, one adding a tryptophan at the hexa-his tag linker (to produce MGWSHHHHHH), and the other introducing a stop codon at position E 102 STOP in the C-terminal domain to create the desired truncations. As *Mt*MscL naturally lacks tryptophan, this residue was added to facilitate quantitation of this protein by the UV absorption at 280 nm. The constructs were inserted into a pET 19b vector carrying ampicillin resistance and transformed into *E. coli* BL21 DE3 *mscL-*, which was the strain previously developed for over-expression of MscL for structural and biochemical studies^17^. Protein was overexpressed by adding isopropyl β-D-1-thiogalactopyranoside (IPTG) to 1 mM once the culture reached an OD_600nm_ of 2–2.5 in Terrific-Broth. After harvesting, cells were lysed using osmotic downshock. Cells were suspended in high osmolarity buffer (50 mM Tris HCl pH 7.5, 200 mM NaCl, 1 mM EDTA, 0.5 μg/mL lysozyme) at a 10 mL buffer per gram of cells ratio with stirring at 4 °C for 1 hour. Thereafter, cells were harvested by centrifugation at 6000 RPM using a JLA 16.250 rotor at 4 °C for 45 minutes, the cell pellet was then quickly re-suspended in low osmolarity buffer at a 10 mL buffer per gram of cells ratio (10 mM Tris HCl, pH 7.5, 20 mM NaCl, 1 mM MgCl_2_, 0.05 µg/mL DNase) at 4 °C for 1 hour to lyse the cells. The lysed cells were harvested by centrifugation at 12500 RPM using a JLA 16.250 rotor at 4 °C for 45 minutes, and then solubilized by homogenizing the pellet and stirring with 20 mM Tris HCl pH 7.5, 100 mM NaCl, 30 mM imidazole pH 8.0, 1% n-dodecyl-β-D-maltopyranoside (DDM) overnight at 4 °C. Cellular debris was removed by ultracentrifugation using rotor Type 45 Ti at 31,500 RPM for 1 hour at 4 °C. MscL constructs were purified using Ni-column affinity chromatography with Qiagen Superflow NiNTA resin (2 mL of a 50% solution of resin per 100 mL of lysate) using the following buffers – Equilibration (5 column volumes): 20 mM Tris HCl pH 7.5, 150 mM NaCl, 10 mM imidazole, 0.05% DDM, Wash (1 column volume): 20 mM Tris HCl pH 7.5, 500 mM NaCl, 25 mM imidazole, 0.05% DDM, Elution (3 column volumes): 20 mM Tris HCl pH 7.5, 150 mM NaCl, 300 mM imidazole, 0.05% DDM. This was followed by Source 30 S anion exchange chromatography using the following buffers – Low salt: 20 mM Tris HCl pH 7.5, 20 mM NaCl, 0.05% DDM, High salt: 20 mM Tris HCl pH 7.5, 1 M NaCl, 0.05% DDM, using a gradient of low salt and high salt buffers for elution. The final step was size exclusion chromatography through a Superdex 200 16/60 column, run with 20 mM Tris HCl pH 7.5, 150 mM NaCl, 0.05% DDM. The purified protein was concentrated with a molecular weight cutoff filter of 50 kDa to a final concentration of 25 mg/mL, as measured by UV_280 nm_ readings using extinction coefficient Abs 0.1% = 0.649.

### Crystallization and structure determination

Crystallization trays were set up using both sitting drop and hanging drop vapor diffusion at 4 °C. *Mt*MscLΔC crystallized in 2 µL + 2 µL drops of protein and crystallization solution. The crystallization solution contained 0.1 M Tris HCl pH 8, 0.15 M CaCl_2_, 25% PEG 400, with 10% of 1 M glycine as an additive directly added to the crystallization drops. After 1.5 weeks, crystals typically grew to 0.4 × 0.3 × 0.1 mm^3^ and were cryo-protected using PEG 400. To provide phase information and to help validate the structure determination, full size crystals were soaked for one week with 5 mM sodium aurothiosulfate (used as a heavy atom derivative in the original MtMscL structure determination). Following soaking, the crystals were briefly crosslinked by adding a 2 µL drop of 25% glutaraldehyde next to the crystal drop and allowing for a 5-minute equilibration period. The derivatized crystals were then harvested and cryoprotected. The native crystals were cryoprotected with 40% PEG 400, whereas the gold derivative crystals were cryoprotected with a mixture of 12.5% diethylene glycol, 25% ethylene glycol, 12.5**%** 2-methyl-2,4-pentanediol, 12.5% glycerol, 12.5% 1,2-propanediol, 12.5% nondetergent sulfobetaine, 3-(1-pyridino)-1-propane sulfonate from the Molecular Dimensions CryoProtX kit. All diffraction data was collected at SSRL BL 12-2 on a Pilatus 6 M detector.

Data processing and refinement were conducted using the CCP4 and Phenix programming suites^38,39^. Diffraction datasets were processed using XDS-GUI and scaled using AIMLESS in the CCP4i suite. The structure was solved by molecular replacement using Phaser MR in CCP4i, and MR-SAD and SAD phase information was obtained from the gold-derivative dataset using Phaser in the Phenix suite. Structure refinement utilized REFMAC5 in CCP4i and Phenix.refine in Phenix^39,53^. Helix crossing angles were calculated with the PyMOL extension, AnglesBetweenHelices. Data collection details and refinement statistics are found in Table 1.

### Permeation pathway analysis

The permeation pathways of the available MscL structures were quantitatively characterized with the program HOLE^42^, with calculated conductances and R_min_, the effective minimal radius, evaluated in default mode. As an alternative measure, the cross-sectional area at narrowest constriction of the permeation pathway was determined by summing the number of points inside the channel on a 1 Å × 1 Å grid on a plane perpendicular to the pore; to approximate the van der Waals surface of pore, all points in the channel were at least 2.8 Å from the nearest protein atom. To provide an empirical calibration between conductance and pore geometry, four proteins with ~nS conductances in 1 M KCl were used as a reference set:

- *E. coli* OmpF (PDB 2OMF, conductance^43^ and structure^54^).
- *Rhodopseudomonas blastica* porin (PDB 1PRN, conductance^55^ and structure^44^).
- mouse Voltage Dependent Anion Channel (VDAC1) (PDB 3EMN, conductance and structure^45^).
- open state MscS (PDB 2VV5, conductance^48^ and structure^46^).

To correct for the effect of salt concentration, the measured MscL conductances were corrected to ~1 M KCl as described in the text.

### Patch Clamp Electrophysiology

Giant spheroplasts were prepared using established protocols^56^, with several important modifications. Briefly, a culture of *E. coli* BL21 DE3 Δ*mscL* containing the construct of interest was treated with cephalexin for 1.5 hours and induced with 1 mM IPTG for 30 minutes. Spheroplasts were then prepared by lysozyme treatment at room temperature for 18–20 min. The spheroplast suspension was centrifuged through 7 mL column of 1 M sucrose at 4 °C and re-suspended in 300 µL 1 M sucrose. Aliquots were stored at −80 °C.

Patch-clamp experiments were carried out using pipette buffer (200 mM KCl, 90 mM MgCl_2_, 5 mM CaCl_2_, 5 mM HEPES, pH 7.4) and bath buffer (200 mM KCl, 90 mM MgCl_2_, 5 mM CaCl_2_, 5 mM HEPES, pH 7.4, 450 mM Sucrose). Excised inside-out patches from spheroplast membranes clamped at −20 mV membrane potential were treated with 5-second symmetric triangle pressure ramps of amplitudes from −50 to −290 mm Hg, using pipettes with bubble number of about 4.5, as previously described^57^. A high-speed pressure clamp system, HSPC-1 (ALA Scientific), was utilized in the experiments. Data were acquired with an Axopatch 200B amplifier and a Digidata 1440 digitizer (Molecular Devices) at 20 kHz, filtered at 5 kHz, and further analyzed with the pCLAMP 10.6 software suite (Molecular Devices). Unitary conductances of MscL channel variants were corrected for pipette access resistance.

## Electronic supplementary material

Supplementary Information
