# Supplementary material for: Elucidating a role for the cytoplasmic domain in the Mycobacterium tuberculosis mechanosensitive channel of large conductance
Source: Sci Rep. 2018 Oct 1;8:14566. doi: 10.1038/s41598-018-32536-6 (PMC6167328; doi:10.1038/s41598-018-32536-6)
Supplement: Supplementary file 1 — Supplementary Information [file 41598_2018_32536_MOESM1_ESM.pdf]

Supplementary Information

Elucidating a role for the cytoplasmic domain in the *Mycobacterium tuberculosis* mechanosensitive channel of large conductance

Nadia Herrera[1], Grigory Maksaev[2], Elizabeth S. Haswell[2], Douglas C. Rees[1,*]

[1]Division of Chemistry and Chemical Engineering

Howard Hughes Medical Institute

California Institute of Technology

Pasadena, CA 91125

[2]Department of Biology

NSF Center for Engineering Mechanobiology

Washington University in St. Louis

St. Louis, MO 63130

*to whom correspondence should be addressed,

email: dcrees@caltech.edu, phone: 626-395-8393

Supplemental Figure S1

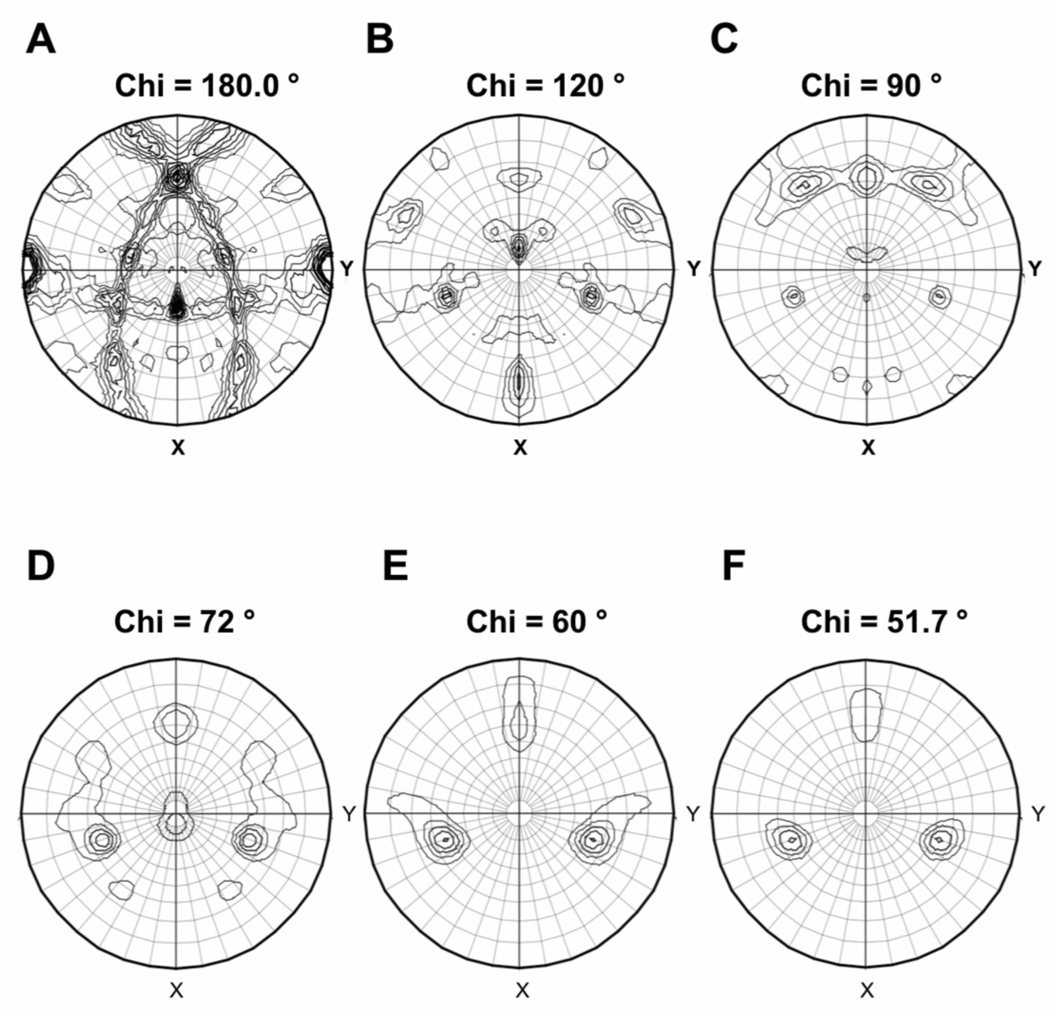

Figure S1. Rotation functions calculated for 2-, 3-, 4-, 5-, 6-, and 7-fold symmetry for the sodium aurothiosulfate (gold) soaked *Mt*MscL∆C crystals (A-F, respectively).

Maps were calculated at 8 Å resolution using the Molrep program in CCP4i. Angles are Chi = 180°, 120°, 90°, 72°, 60°, and 51.7°. All the maps were calculated with the same 180˚, 120˚, and 90˚ sections to represent equal contours.

Supplemental Figure S2

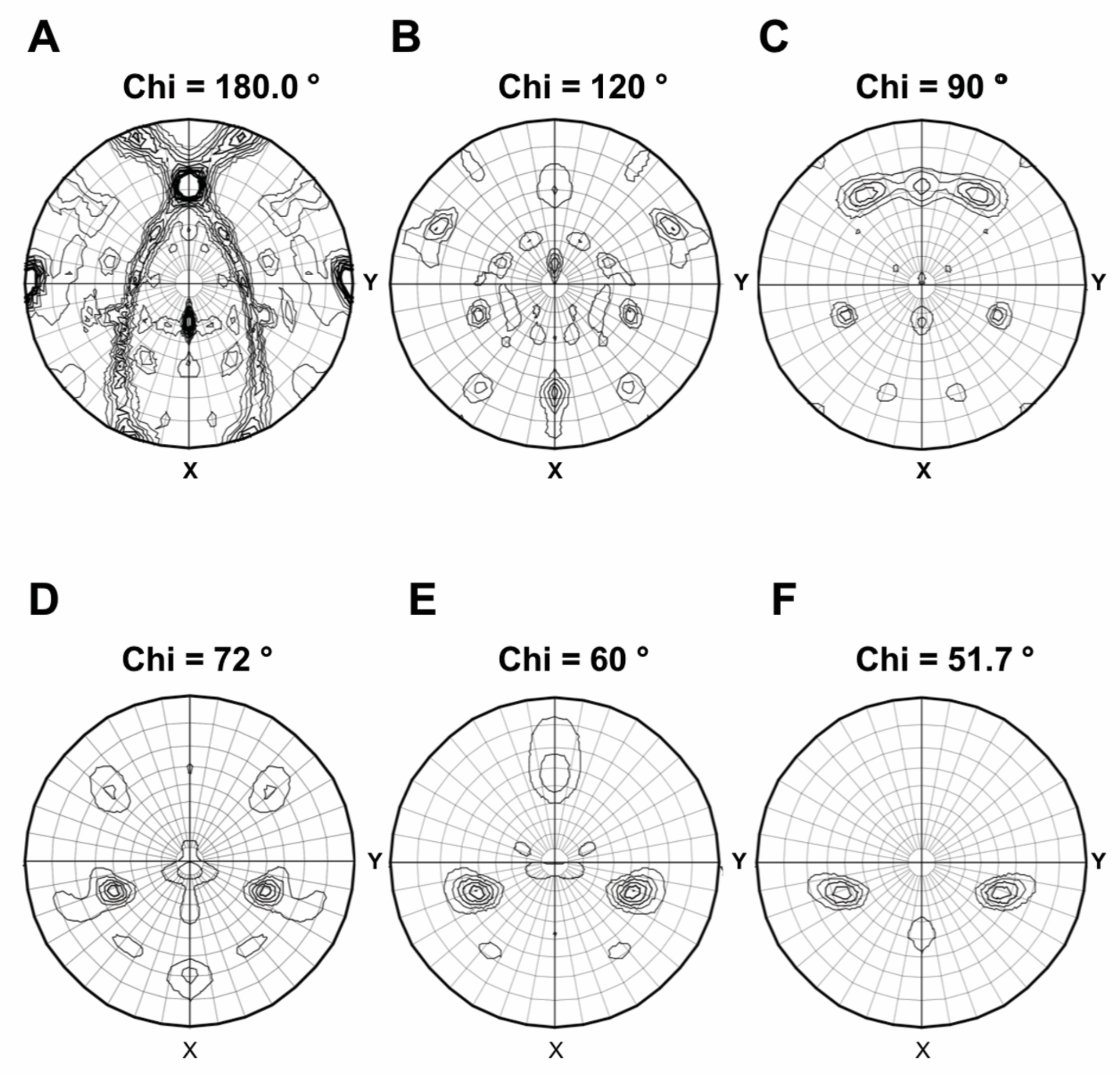

Figure S2. Rotation functions calculated for 2, 3, 4, 5, 6, and 7 fold symmetry for Native *Mt*MscLΔC crystals (A-F, respectively).

Maps were calculated at 8 Å resolution using the Molrep program in CCP4i. Angles are Chi = 180°, 120°, 90°, 72°, 60°, and 51.7°. All the maps were calculated with the same 180˚, 120˚, and 90˚ sections to represent equal contours.

Supplemental Figure S3

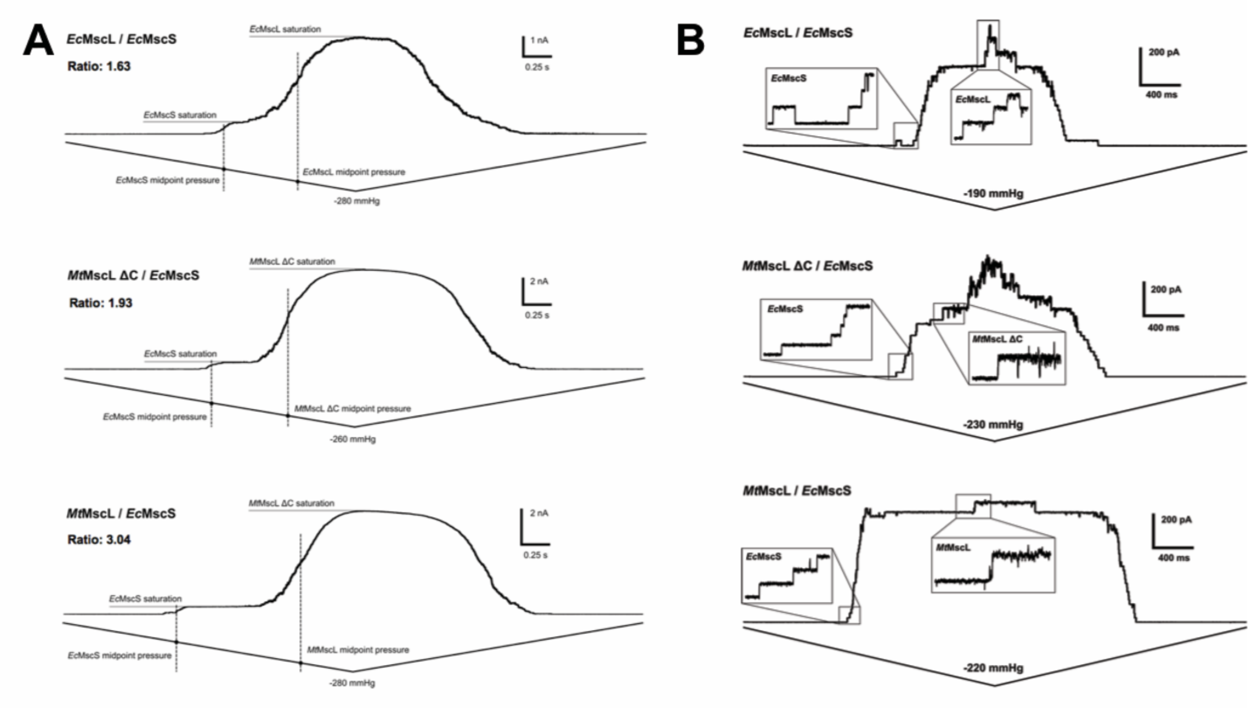

Figure S3. Examples of data used to generate Figure 5 in manuscript.

(A) Representative traces of tension (or pressure) induced currents in patches derived from BL21 DE3 Δ*mscL* cells expressing *Ec*MscL, *Mt*MscL ΔC, or *Mt*MscL full-length. The midpoint pressure for *Ec*MscS and the relevant MscL variant is indicated, as well as the point on the curve where current saturation is achieved. (B) Representative single channel traces for *Ec*MscL, *Mt*MscL ΔC, and *Mt*MscL. Gating events were observed in all of the patches shown upon application of the triangular pressure ramp (suction to the pipette) of the indicated amplitude (mm of mercury). The suction applied to the patch pipette in each experiment is indicated underneath the trace and the maximum value of negative pressure reached is indicated.

Supplemental Figure S4

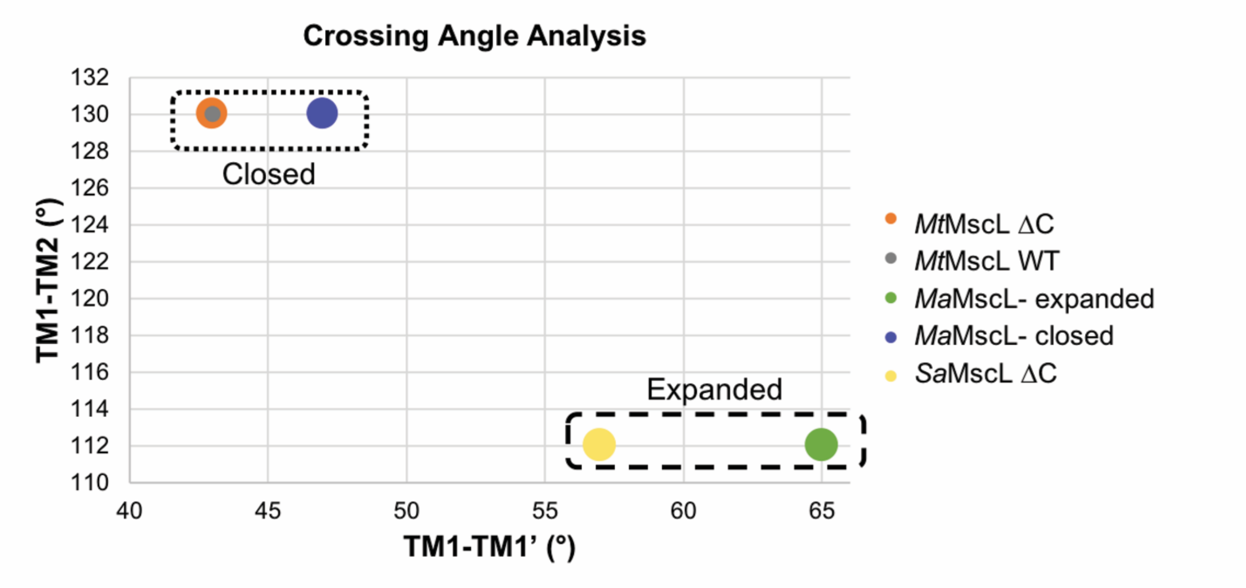

Figure S4. Crossing angle analysis of MscL structures.

A categorization of the conformational states of MscL can be made by plotting the helix-helix crossing angles between TM1-TM1' and TM1-TM2. Structures in the closed or expanded states can be differentiated by the characteristic relationships between these crossing angles in the two states. TM1-TM1' is plotted on the X axis and TM1-TM2 is plotted on the Y axis, values are represented in degrees.
